# Some Military Medical Economies

**Published:** 1916-02-12

**Authors:** 


### SOME MILITARY MEDICAL ECONOMIES.

### The Value of Criticism.

There are signs that the exhortations to greater thrift and improved organisation are receiving the attention of the military medical authorities. It may be they have learned that the medical recruiting campaign is hindered by the criticisms of those who urge that the Army is not using wisely the services of those who have already taken commissions in the R.A.M.C. Perhaps, also, they have read the recent correspondence in the columns of the *Daily Telegraph*, and have been convinced that improvements are possible. When a statement is made that at a certain hospital there are more "colonels" than patients, it is time the question was investigated, even though some allowance be made for an element of dramatic exaggeration in the description. Whatever the cause, it is a matter for satisfaction that in the midst of their difficulties—and these, indeed, have been neither light nor few—the authorities have found time to attend to certain matters which have been brought to light by the influence of outside criticism. Here we have always recognised that such criticism should be helpful and not hostile, and that due regard should be paid to the exceptional circumstances of the hour and to the heavy burdens which those in authority have to bear. It is the plain duty of all who have influence on public opinion to keep that opinion steadfast and resolved, and this is to be aided not by the cultivation of sensational discoveries but by the presentation of the various issues in such a fashion as to secure that no reasonable complaint or hardship shall be overlooked. On this plane criticism and examination have profitable scope, and are welcomed and encouraged by all who desire to unite and concentrate a maximum national endeavour. To conquer the foe who is without is the first consideration, and to avoid grievances at home is an aid to this end.

In a special measure this Journal is concerned with the interests of the civil hospitals and with the constituencies which they serve. It is under this responsibility that we have on more than one occasion felt compelled to urge that the War Office was not using the hospitals quite fairly. Readily the managers of the hospitals placed a large proportion of their resources at the disposal of the military authorities, and as a mattter of fact a very large number of sick and wounded soldiers have been treated at these institutions. Inevitably this has meant prejudice to the civil population, and though on the ground of principle this proceeding could hardly be defended, we have always recognised that in the special circumstances of the day no other course was possible, and that public opinion was prepared to justify it. To be quite candid, we have thought that the generosity of the civil hospitals was in some degree abused, and that the War Office ought to have depended more on their own organisation. More urgently, it may be remembered, we have pointed out that numbers of the soldiers sent to the civil hospitals were not really in need of hospital treatment, and that many who had recovered were still retained in the wards. This was, and is, a manifest abuse which hospital managers ought not to admit. With these latter rest the interests of the sick poor among the civil population, and it cannnot be just to use as mere resting places or convalescent homes institutions which are organised and supported for the relief of serious illness. We have repeated this argument again and again because there was an obvious danger that under a patriotic sentiment a real injustice might fail to be recognised. Hence it is with much satisfaction we learn that the War Office has given attention to the subject, and that a steady attempt is to be made to redress the grievance. The civil hospitals, we understand, are no longer to be asked to shelter convalescent patients, who, on the contrary, as soon as they are fit to leave the hospitals, are to be discharged to the care of the military authorities. Presumably they will be drafted to military institutions in country districts where the conditions are favourable to a complete restoration of their health. The result, of course, will be that many beds will in this way be set free for the purpose for which they were intended—namely, the medical and surgical care of the sick poor. In regarding this as good news, it is, we are sure, unnecessary for us to say that we grudge no good thing to the men who have fought and suffered for the cause of the nation. Theirs is certainly the first claim on us all. But we have striven to satisfy this claim without inflicting injustice and hardship on others, and naturally we are gratified that this point of view has received practical appreciation. The position is all the more satisfactory when we recollect that for the soldiers themselves convalescent facilities in the country are more favourable than those which exist in the hospital wards of our big towns.

Another evidence of War Office economy is to be seen in the changes made in regard to the holders of *à la suite* commissions. These gentlemen are for the most part physicians and surgeons of the civil hospitals, who receive rank and pay since the date of mobilisation and are in charge of the medical and surgical services of the military hospitals in the towns in which they reside. It has been freely said that many of these hospitals have been much overstaffed, and in the London district at least a reduction is at once to be practised. The arrangement now to obtain is that at each of the general base hospitals the work is to be done by the resident staff, while the visiting staff is to consist of a limited number of physicians and surgeons—usually two physicians and three surgeons. Only in the event of an increase in the number of patients are additional members of the staff to be summoned for active duty, and the summons will be dictated by considerations of seniority. The arrangement will not perhaps be appreciated by some of the officers concerned, and it is a little difficult to conceive what will be the exact military status and pay of one who either has no duties or only contingent duties. But

(*Continued on page 432.*)

### SOME MILITARY MEDICAL ECONOMIES—(*continued from page* 431).

the alteration does show that the War Office is alive to some of the errors of the present situation, and that, so far as possible, these shall be corrected. The new rule means that more practitioners are restored to the responsibilities of civil practice, and if their military labels disappear we venture to think their dignity will not suffer. In some instances, it appears, the holders of the *à la suite* commissions have received notice that certain allowances hitherto paid to them will not be continued. Quietly serving their country in the cities and towns where they have long been wont to reside, it seems that these officers have not taken to tents but have lived peacefully in their own homes. The War Office has discovered this astonishing fact, and hence the allowances are to be docked.

